# First Genome Sequence of a Canine Distemper Virus Strain from South America

**DOI:** 10.1128/genomeA.01009-14

**Published:** 2014-10-23

**Authors:** Nicolás Sarute, María V. Delgado, Lucía Carrau, Alejandro Benech, Lourdes Francia, Ruben Pérez, Yanina Panzera

**Affiliations:** a Sección Genética Evolutiva, Instituto de Biología, Facultad de Ciencias, Universidad de la República, Montevideo, Uruguay; b Departamento de pequeños animales, Facultad de Veterinaria, Universidad de la República, Montevideo, Uruguay

## Abstract

Canine distemper virus causes a severe infectious disease in carnivores worldwide. Herein, we sequenced and analyzed the genome of a new strain (Uy251/2012) isolated from a dog in Uruguay. The Uy251/2012 strain belongs to the Europe1/South America1 lineage, and constitutes the first report of a genomic sequence in South America.

## GENOME ANNOUNCEMENT

Canine distemper virus (CDV) belongs to the *Morbillivirus* genus, *Paramyxoviridae* family, and possesses a nonsegmented single-stranded negative RNA genome that encodes six structural proteins (1). CDV is the etiological agent of distemper, a severe systemic disease that affects domestic and wild carnivores worldwide. The disease is characterized by several clinical signs (respiratory, gastrointestinal, and/or central nervous system) and high mortality rates (2).

For decades, the disease was controlled using attenuated vaccines; however, CDV outbreaks are constantly reported in dogs and wildlife, with an increase in the number of infected dogs, both vaccinated and unvaccinated (3).

CDV strains are classiﬁed into geographic lineages based on the variability of the hemagglutinin (H) and the fusion protein signal-peptide (Fsp) (4, 5).

Herein, we amplified the complete coding and intergenic regions of the Uy251/2012 South American strain obtained from a dog’s necropsy. The viral RNA was isolated from a lung slice (~150 mg) using Trizol reagent (Invitrogen) according to the manufacturer’s instructions; the ﬁrst-strand cDNA was synthesized using random primers (Invitrogen). Nine pairs of primers were designed based on the reference sequence CDV 5804 strain (AY386315). Phusion High-Fidelity DNA Polymerase (Thermo Scientific) was used to amplify the genome in nine overlapping fragments. Puriﬁed amplicons were sequenced bidirectionally using an ABI3130 genetic analyzer (Applied Biosystems). Sequences were compiled and edited using the SeqMan program (Lasergene). The coding genome of the Uy251/2012 strain consists of 15,447 bp and encompassed six genes (3′ N–P-M-F–H-L 5′). The Uy251/2012 strain genome was compared with 40 complete genomes available at the Genbank database belonging to six CDV lineages previously described. Based on the phylogenetic analysis (maximum likelihood–general time reversible model, Gamma distribution), the Uy251/2012 strain was grouped within the Europe1/South America1 lineage (EU1/SA1), as reported for all the Uruguayan strains previously analyzed (5–7). The nucleotide and amino acid (nt/aa) identity within this lineage was 98.2/98.8%, respectively. The mean nt/aa identities between the EU1/SA1 and other lineages were 95.9/96.3% for North America1, 94.8/95% for Asia1, 94.5/94.9% for Asia2, 94.9/93.2% for North America2, and 94.2/94.2% for Europe3. The comparison with the vaccine strains showed lower values of nt/aa identity of 92.5/92.6%, respectively.

When comparing each individual gene the following values for nt/aa identities were obtained: 95.7/97.5% (N gene), 95.6/94.3% (P gene), 95.5/98.2% (M gene), 92.8/91.7% (F gene), 92.4/92.1% (H gene), and 94.6/85.3% (L gene). These values are in agreement with previous studies that established that the H and F genes are highly variable (4, 8, 9); strikingly, the highest amino acid divergence was detected for the L protein, which has not been analyzed for CDV field strains to date.

The Uy251/2012 strain genome sequence is the first report for South America so far. The high genetic variability between Uy251/2012 and vaccine strains should be considering for the formulation of new and updated vaccines for South America. Further analysis, including more genomic sequences, will contribute to the knowledge of the CDV evolutionary landscape in this region.

### Nucleotide sequence accession number.

This sequence was deposited at GenBank under the accession number KM280689.
